# Using Smartphone Survey and GPS Data to Inform Smoking Cessation Intervention Delivery: Case Study

**DOI:** 10.2196/43990

**Published:** 2023-06-16

**Authors:** Amanda Luken, Michael R Desjardins, Meghan B Moran, Tamar Mendelson, Vadim Zipunnikov, Thomas R Kirchner, Felix Naughton, Carl Latkin, Johannes Thrul

**Affiliations:** 1 Department of Mental Health Johns Hopkins Bloomberg School of Public Health Baltimore, MD United States; 2 Spatial Science for Public Health Center Johns Hopkins University Baltimore, MD United States; 3 Department of Epidemiology Johns Hopkins Bloomberg School of Public Health Baltimore, MD United States; 4 Department of Health, Behavior and Society Johns Hopkins Bloomberg School of Public Health Baltimore, MD United States; 5 Department of Biostatistics Johns Hopkins Bloomberg School of Public Health Baltimore, MD United States; 6 Department of Social and Behavioral Sciences New York University School of Global Public Health New York, NY United States; 7 Center for Urban Science and Progress New York University Tandon School of Engineering New York, NY United States; 8 Behavioural and Implementation Science Research Group University of East Anglia Norwich United Kingdom; 9 Sidney Kimmel Comprehensive Cancer Center Baltimore, MD United States; 10 Centre for Alcohol Policy Research La Trobe University Melbourne Australia

**Keywords:** adult, application, case study, cessation, delivery, GIS, GPS, health interventions, mHealth, mobile phone, smartphone application, smartphone, smoker, smoking cessation, smoking

## Abstract

**Background:**

Interest in quitting smoking is common among young adults who smoke, but it can prove challenging. Although evidence-based smoking cessation interventions exist and are effective, a lack of access to these interventions specifically designed for young adults remains a major barrier for this population to successfully quit smoking. Therefore, researchers have begun to develop modern, smartphone-based interventions to deliver smoking cessation messages at the appropriate place and time for an individual. A promising approach is the delivery of interventions using geofences—spatial buffers around high-risk locations for smoking that trigger intervention messages when an individual’s phone enters the perimeter. Despite growth in personalized and ubiquitous smoking cessation interventions, few studies have incorporated spatial methods to optimize intervention delivery using place and time information.

**Objective:**

This study demonstrates an exploratory method of generating person-specific geofences around high-risk areas for smoking by presenting 4 case studies using a combination of self-reported smartphone-based surveys and passively tracked location data. The study also examines which geofence construction method could inform a subsequent study design that will automate the process of deploying coping messages when young adults enter geofence boundaries.

**Methods:**

Data came from an ecological momentary assessment study with young adult smokers conducted from 2016 to 2017 in the San Francisco Bay area. Participants reported smoking and nonsmoking events through a smartphone app for 30 days, and GPS data was recorded by the app. We sampled 4 cases along ecological momentary assessment compliance quartiles and constructed person-specific geofences around locations with self-reported smoking events for each 3-hour time interval using zones with normalized mean kernel density estimates exceeding 0.7. We assessed the percentage of smoking events captured within geofences constructed for 3 types of zones (census blocks, 500 ft^2^ fishnet grids, and 1000 ft^2^ fishnet grids). Descriptive comparisons were made across the 4 cases to better understand the strengths and limitations of each geofence construction method.

**Results:**

The number of reported past 30-day smoking events ranged from 12 to 177 for the 4 cases. Each 3-hour geofence for 3 of the 4 cases captured over 50% of smoking events. The 1000 ft^2^ fishnet grid captured the highest percentage of smoking events compared to census blocks across the 4 cases. Across 3-hour periods except for 3:00 AM-5:59 AM for 1 case, geofences contained an average of 36.4%-100% of smoking events. Findings showed that fishnet grid geofences may capture more smoking events compared to census blocks.

**Conclusions:**

Our findings suggest that this geofence construction method can identify high-risk smoking situations by time and place and has potential for generating individually tailored geofences for smoking cessation intervention delivery. In a subsequent smartphone-based smoking cessation intervention study, we plan to use fishnet grid geofences to inform the delivery of intervention messages.

## Introduction

### Cigarette Smoking and Smoking Cessation Among Young Adults

Although cigarette use has declined in recent decades, of a weighted sample of 4200 young adults (ages 19-30 years), 491 (11.7%) young adults in the United States reported current (past 30-day) cigarette smoking in 2019 [1]. Smoking cessation interventions can provide societal and health care cost savings, as well as immediate and long-term health benefits for young adults (eg, decreased risk of cardiovascular diseases, chronic obstructive pulmonary disease, and several types of cancer) [2].

Young adults who smoke rarely use evidence-based smoking cessation strategies in their quit attempts. We need novel interventions that can reach young people and help them quit smoking. A recent study using data from the Population Assessment of Tobacco and Health found that almost all young adults who smoke would like to quit at some point in their lives, but few young adults with a recent quit attempt relied on evidence-based cessation strategies [3]. One explanation for this may be that many interventions for young adults attempt to prevent smoking initiation rather than support smoking cessation [4].

### Mobile Phones for Smoking Cessation

The ubiquity of smartphones may help enhance the feasibility, acceptability, and reach of smoking cessation interventions. Almost all 18- to 29-year-olds in the United States own a smartphone [5]. As a result, GPS-enabled smartphones allow researchers to study the behaviors, mobility, and activity spaces of individuals and deliver mobile health (mHealth) interventions that were previously not feasible for potential consumers to access [6]. However, the efficacy of mHealth is understudied in many areas of public health, including smoking cessation.

Few interventions with location information (eg, GPS) include formal spatial science components that may improve intervention delivery. Smoking is often a geographically triggered behavior; people may regularly smoke in the same locations (eg, bars) or have cravings due to an environmental exposure (eg, product or advertisement exposure in a convenience store) [7,8]. GPS-enabled smartphones can register when an individual is near or at a location at-risk for smoking and deliver just-in-time cessation support to resist environmental triggers [9].

Spatial methods are essential for improving smoking cessation by examining the nexus between health and place [8,10,11]. For example, 1 study developed a deep learning model to predict smoking events based on GPS smartphone data [12]. The authors were able to predict smoking events accurately on weekdays and weekends (mean 0.87, SD 0.08) using a 1D convolutional neural network [12]. Overall, the literature using fine-grained geographic information to inform smoking cessation intervention delivery on smartphone apps is scarce, and this proof-of-concept study aims to address this gap.

### Geofences for Smoking Cessation

Geofences are virtual perimeters or zones that can trigger smartphone notifications for individuals when entering, exiting, or dwelling within a specified geographic area [13]. Geofences may benefit mHealth interventions since individuals can receive interventions at high-risk locations [14]. Using participants’ mobility patterns to generate geofences with the goal of promoting positive behavior change is a growing area of interest in geography and public health research [15,16]. For example, geofencing applications have been developed to support dentist accessibility [17], gambling cessation [18], awareness of air pollution exposure [19], COVID-19 surveillance [20], and tobacco retail exposure for smoking cessation [8], among other uses.

In the context of smoking cessation, Naughton et al [9] studied a cohort of 15 individuals in the United Kingdom and disseminated geofenced-triggered messages to participants when they entered high-risk smoking zones (ie, circular zones with a 100 m radius containing at least 4 self-reported smoking events); the study, however, did not use formal spatial analytical techniques to generate geofences. To extend this previous research, our goal for this study is to generate geofences around high-risk locations for smoking using a kernel density estimation (KDE) approach, which is reliable for analyzing GPS-based activity space data [21]. KDE is a proven spatial method that identifies hot spots of point patterns in space and time. Although other point-pattern techniques are available, KDE is very flexible regarding its parameter customization (eg, bandwidth, output resolution of the density surface, and kernel functions), which is described more in the Methods section [22]. However, the literature on using KDE approaches to generate high-risk geofences is very limited (eg, transportation injury prevention [23,24]) and has been underused for creating geofences tailored to individuals’ spatiotemporal patterns of health risk behaviors.

Additionally, the uncertain geographic context problem (UGCoP) poses a challenge for geofence construction [25]. UGCoP represents the concern that the geographic units used for analyses may not represent the “true causally relevant” geographical context [25]. UGCoP is an issue for geofence construction because the geofenced location influences a person’s smoking behaviors across space and time, but as presented in UGCoP, the appropriate spatial and temporal dimensions for geofence construction are uncertain. Individuals often report smoking at home, outside, or in the car [26], which could constitute the “true causally relevant” geographic context [25], but few interventions sensitive to capturing the “true causally relevant” geographic context have been developed or tested.

### Research Objectives and Anticipated Contributions

This study uses self-reported smartphone surveys and passively tracked GPS data collected from young adults who smoke. The objective is to develop a spatial analytical approach to identify hot spots of self-reported smoking events and to produce KDE-informed geofences for catered smoking cessation intervention delivery in future studies. The proposed method, which incorporates spatial methods, may be applicable to intervene on other health conditions or other substance use as well. In this study, we demonstrate an exploratory method of generating person-specific geofences for high-risk smoking areas by presenting 4 case studies. We chose to test this method on 4 participants as a case study rather than the entire sample to examine the nuances of this methodological approach that would be more difficult to observe within the entire sample. By focusing on 4 participants, we can identify individual-level variation in smoking behaviors and locations that may influence geofence construction and performance. More importantly, we can better understand why this method may not have worked for certain situations or individuals. Although these 4 participants do not capture the full variation of smoking behaviors, they provide an important first step to evaluating this method in light of unanticipated situations and circumstances. For the 4 cases, we create individually tailored geofences that vary temporally, accounting for behavioral changes over the course of a day. A KDE-informed approach can capture the intersection of place and health by taking a person-centered, data-driven approach. To address UGCoP, we experiment with time-specific geofences constructed by various geographical zones and assess how well the different geofences capture smoking events for each case. We operationalize geofence performance as the percent of smoking events captured, such that an ability to capture greater than 80% of smoking events within geofences for a particular time frame was considered good, while greater than 50% was considered adequate.

## Methods

### Study Design

Young adults from Alameda and San Francisco counties participated in an ecological momentary assessment (EMA) study for 30 days that captured individual-level, spatiotemporal patterns of smoking behaviors. Demographic information, smoking history, and alcohol use were assessed through Qualtrics [27] at baseline. For 30 days, participants reported smoking events and completed 3 daily surveys distributed randomly throughout the day through the PiLR Health app [28]. In the app, participants recorded if they were about to smoke (ie, a smoking event), after which some were asked to complete a smoking survey based on the average number of daily cigarettes smoked at baseline (eg*,* a baseline rate of 10 cigarettes per day was associated with a 33% chance of receiving a smoking survey). To minimize the burden of participation, smoking surveys were limited to at most 3 per day. Each submitted survey was date-, time-, and GPS-stamped. See previous publications for more information on data collection procedures [29-31].

We selected 4 participants for the case study based on their overall compliance with EMA data collection procedures. The total available data, which includes both smoking and nonsmoking reports, served as a proxy for compliance. We selected 4 case study participants from the total available data quartiles. This sampling strategy was chosen to investigate the feasibility of geospatial analyses for participants with different rates of EMA self-report compliance and to improve the generalizability of findings.

### Ethics Approval

All study procedures were approved by the San Francisco Committee on Human Research, University of California (15-18033).

### Participants

Eligible participants were recruited using Facebook and Instagram advertisements between 2016 and 2017, were between 18 and 26 years of age, were established smokers (ie, at least 100 cigarettes per lifetime), and reported currently smoking at least one cigarette per day on at least 3 days per week. Study eligibility also required daily smartphone use with GPS capabilities. Women identifying as a sexual minority were oversampled for a nested qualitative study [29]. Study consent was provided electronically on Qualtrics. To confirm their identity, participants were required to send a photo of their ID.

### Measures

#### Smoking Events

The outcome of interest was self-reported cigarette smoking events (yes or no). EMA data included smoking events (eg, cigarette self-reports) and nonsmoking events (eg, random surveys with nonsmoking events) with a linked GPS location. Smoking events reported within 5 minutes of another smoking event were dropped to correct for measurement errors due to technical difficulties with the app. GPS locations were converted to North American Datum 1983 California Zone 3 in US feet.

#### Time

EMA data included time stamps that were collected in Coordinated Universal Time (UTC) and converted to Pacific Time for analyses. Time was categorized into 3-hour periods (eg, midnight-2:59 AM, 3 AM-5:59 AM, 6 AM-8:59 AM, and 9 AM-11:59 AM).

#### Baseline Demographics

Demographic data (eg, age, gender, race or ethnicity, and education) were collected at baseline. The frequency of past 30-day cigarette use was also obtained.

### Spatial Analyses

#### Framework of KDE

We employed KDE to identify high-risk zones for each individual. In other words, KDE was run separately for each participant to effectively tailor the resulting geofences to each individual. KDE is a moving window method that calculates the density as the number of events based on their distance to the center of a circle with a radius of the specified bandwidth *τ,* which determines the degree of spatial smoothing [32]. The window moves and centers along the intersections of a grid and calculates the density at each intersection, which is then considered in unison to provide a weighted average for a location. Events (ie, points) closer to the center of the search radius receive higher weight [33]. The KDE function is defined in Equation 1:



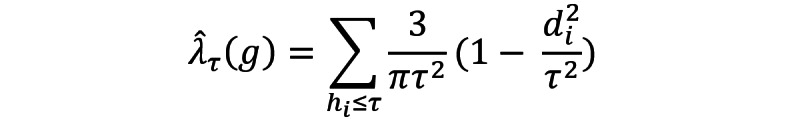



where 
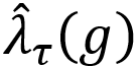
 is the kernel density value at grid point *g*; is the Euclidean distance between grid point *g* and event *i*; and 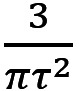 is the weight where its value equals “0” at distance *τ* [34]. To identify high-risk areas for smoking for each participant, we plotted a kernel density plot of smoking events with a bandwidth of 1320 ft (ie, 0.25 miles), a proxy for comfortable walking distance [35], and a raster cell output size of 150 ft^2^. We then extracted high-risk zones using zonal statistics. Zonal statistics calculate a statistic of interest for raster values falling within a “zone” chosen by researchers in another data set [36]. For our study, we averaged KDE raster values within 3 zones: 2020 US census blocks, 500 ft^2^ fishnet grid cells, and 1000 ft^2^ fishnet grid cells. We then minimum-maximum normalized the mean KDE to the range of 0 to 1 and retained zones with normalized mean KDEs above a threshold chosen during sensitivity analyses. To distinguish risk levels within zones, we categorized zones with normalized mean KDEs above the threshold into terciles (ie, low, medium, and high risk) for each case.

To identify the threshold for “high-risk,” we assessed the performance of geofences constructed for normalized mean KDE thresholds of 0.1, 0.2, 0.3, 0.4, 0.5, 0.6, 0.7, 0.8, and 0.9 for the 4 case studies. We identified the high-risk normalized mean KDE threshold at the block-level. Performance was assessed by examining the percent of smoking events captured within geofences, irrespective of time of day.

#### Zonal Statistics by Census Block

We had first chosen census blocks as the “zone” because census blocks are the smallest geographical unit with demographic data and are a federally recognized statistical area [37]. A geographical unit tied to demographic data could be useful for researchers interested in controlling societal-level demographic variables. Further, the census block is a stable geographical unit across the United States with a unique identifier determined by the US Census Bureau. In terms of stability, the census block is reconsidered once every decade [37].

The census block, however, is constructed by both physical (eg, roads, streams, and railroad lines) and nonphysical structures (eg, property lines and city limits), which leads to variability in census block size [37] and thus a normalized mean KDE. Census blocks may be the size of a city block in urban environments or up to hundreds of square miles in rural areas [37]. As a result, differences in census block size may affect block-level normalized mean KDEs (eg, smaller blocks may have fewer raster values to average across than larger blocks). Larger blocks may also have more heterogeneity in raster values than smaller blocks, decreasing the precision of the mean KDE.

Moreover, identifying a large census block as high-risk poses some challenges for intervention delivery. Based on our method, the geofence around the entire census block would trigger the intervention for a large area even though the participant may smoke only in a small subarea of the census block. Census blocks, however, may still offer value in cities where blocks are often equivalent to city blocks and in studies seeking to account for demographic variables [37].

#### Zonal Statistics by Fishnet Grid

Alternatively, we can create uniform zones by overlaying a fishnet grid over the area of interest (eg, Alameda and San Francisco counties). A fishnet grid is an array of square cells fitted within a geographical area [38]. We repeated the same process of finding the normalized mean KDE and labeling high-risk areas as zones with a normalized mean KDE above the threshold chosen from the census block analyses, except that the zone was a cell in the fishnet grid rather than a census block. We created 2 fishnets, one with 500 ft^2^ cells and another with 1000 ft^2^ cells.

#### Geofence Construction

For each 3-hour time interval, high-risk zones were identified as those with (1) normalized mean KDEs above the threshold of 0.3 and (2) at least one smoking event. Normalized mean KDEs were based on all observations of a given participant. For example, each block has the same normalized mean KDE value across all hours of the day and for each day of the week. A block, however, may be high-risk at 1 time of day (eg, evening) yet low-risk at another time of day (eg, morning) only because the participant has no history of smoking during the time of day it is considered low-risk (eg, morning). Once the high-risk blocks were identified, geofences were constructed by generating 100-m buffers around groups of adjacent high-risk blocks or cells. All spatial analyses were conducted in ArcGIS Pro (version 2.8.0) [39].

## Results

### Sample

The population included 144 participants with a mean age of 22.7 (SD 2.6) years at baseline; 76 of 144 (52.8%) were female; and 57 of 144 (39.6%) identified as non-Hispanic White, 31 of 144 (21.5%) as Hispanic or Latino, and 30 of 144 (20.8%) as Asian (Table 1). Most of the population had either completed some college (54/144, 37.5%) or received an associate or bachelor’s degree (51/144, 35.4%). The median number of days with at least one cigarette smoked was 30 (IQR 24-30). On days with at least one smoking event, participants reported smoking a median of 5 (IQR 3-8) cigarettes per day.

**Table 1:** Baseline characteristics.

Characteristics		Cases sampled (N=4)			Cases not sampled (N=140)	Overall (N=144)
Age (years), mean (SD)		21.3 (2.6)			22.7 (2.4)	22.7 (2.5)
**Sex, n (%)**						
	Male	2 (50)			66 (47.1)	68 (47.2)
	Female	2 (50)			74 (52.9)	76 (52.8)
**Highest education, n (%)**						
	Less than or equal to high school	1 (25)			30 (21.4)	31 (21.5)
	Some college		2 (50)	52 (37.1)		54 (37.5)
	Associate or bachelor’s degree	1 (25)			50 (35.7)	51 (35.4)
	Master’s degree or higher	0 (0)			8 (5.7)	8 (5.6)
**Race or Ethnicity, n (%)**						
	Non-Hispanic White	3 (75)			54 (38.6)	57 (39.6)
	Non-Hispanic Black	0 (0)			6 (4.3)	6 (4.2)
	Asian	1 (25)			29 (20.7)	30 (20.8)
	American Indian or Alaska Native	0 (0)			1 (0.7)	1 (0.7)
	Native Hawaiian or Pacific Islander	0 (0)			2 (1.4)	2 (1.4)
	Hispanic	0 (0)			31 (22.1)	31 (21.5)
	Other or Multiracial	0 (0)			17 (12.1)	17 (11.8)
Cigarettes per smoking day, median (range)		3.5 (3-5)			5 (1-30)	5 (1-30)
Smoking days in the past 30 days, median (range)		30 (25-30)			30 (0-30)	30 (0-30)

### Cases Selected for Analysis

Of the 4 cases sampled; their ages ranged from 19 to 25 years, with an average age of 21.3 (SD 2.6) years at baseline. Two were male and 2 were female at birth. Three participants identified as non-Hispanic White and one as Asian. One had less than or equal to a high school education, 2 had some college, and 1 had an associate’s or bachelor’s degree. Three of the cases reported daily smoking in the past 30 days, while one reported smoking on 25 of the past 30 days. The cases reported smoking between 3 and 5 cigarettes per smoking day in the baseline assessment.

The number of smoking events reported by these 4 cases in EMA data ranged from 16 to 177, while the number of nonsmoking events ranged from 8 to 67 (Table 2). All smoking events were in San Francisco and Alameda counties.

**Table 2:** Smoking and nonsmoking events of cases.

Quartile	Smoking events, n (%)	Nonsmoking events, n (%)	Total reports, n (%)
25th	16 (66. 7)	8 (33.3)	24 (5.7)
50th	12 (20.3)	47 (79.7)	59 (14.1)
75th	31 (31.6)	67 (31.6)	98 (23.4)
100th	177 (74.7)	61 (74.4)	238 (56.8)

### Threshold for Identifying High-Risk Census Blocks

**Figure 1:**
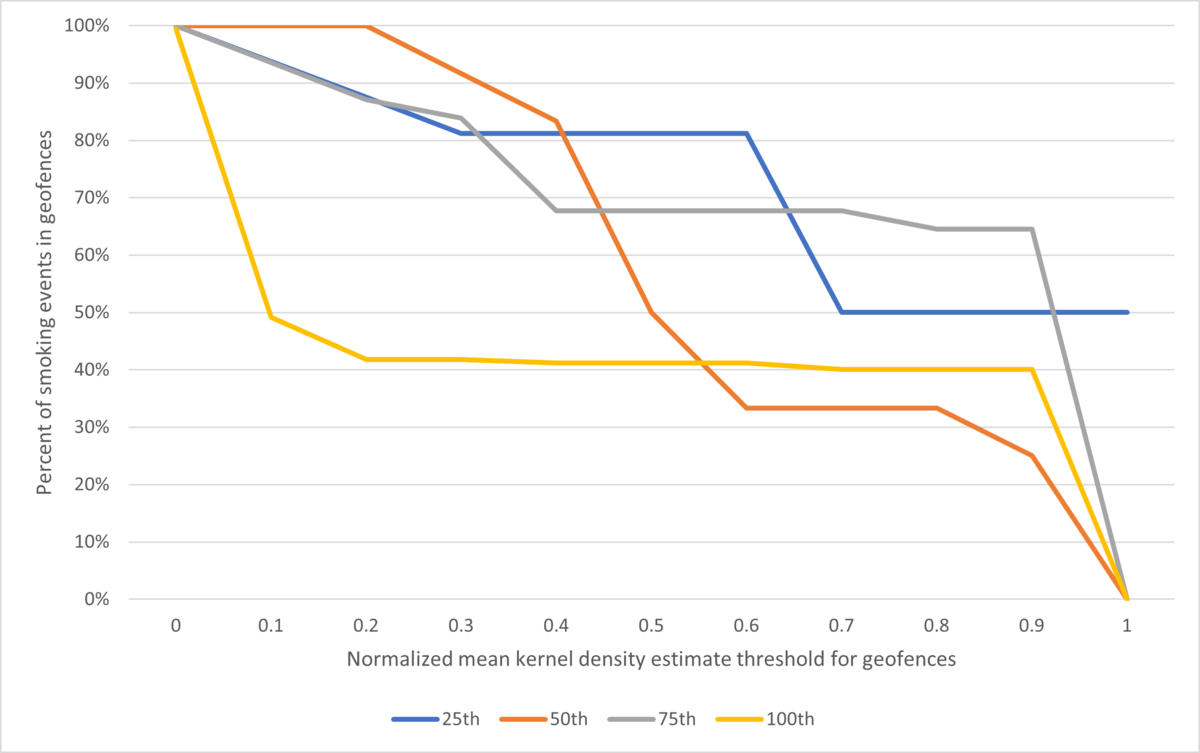
Percent of smoking events within geofences for census blocks among 4 participants at quartiles of ecological momentary assessment self-reported data.

The percent of smoking events captured in time-independent geofences at varying normalized mean KDE thresholds of high-risk for census blocks are presented in Figure 1. The figure displays the percentage of smoking events contained within constructed geofences if zones with normalized mean KDE values above each threshold are retained. For all but 1 participant, a normalized mean KDE threshold of 0.5 captured 50% or more of all possible smoking events within generated geofences.

We proceeded to construct geofences across 3-hour time intervals for census blocks with normalized mean KDEs greater than or equal to 0.3 because they captured at least 80% of smoking events for all but 1 case.

Of note, 3 participants had no geofences generated for census blocks with a normalized mean KDE of 1.0 due to rounding differences (eg, rounding was done to 6 decimal places such that 0.999998 would not be included for this threshold). A 0.0 threshold should capture every block with at least one smoking event reported; however, we had 2 participants that were missing 1 block with at least one smoking event reported in it. This occurred because these smoking events occurred in very small, narrow blocks that were part of roadways, which consequently did not rasterize for a cell output size of 150 ft^2^. In other words, these blocks were missing normalized mean KDE values because the blocks were smaller than the cell’s output size. In some cases, this may have left out a block at risk for smoking. See Table S1 in Multimedia Appendix 1 for summary statistics of the 4 cases’ normalized mean KDEs.

### Comparison of Geofence Construction Methods of Census Blocks Compared to Fishnet Grid Across 3-Hour Time Intervals for Each Case

Across 3-hour time intervals, the average percentage of smoking events within geofences ranged from 36.4% to 100%. Geofences contained the highest percentage of smoking events between midnight and 3 AM and between 9 AM and 11:59 AM. Conversely, geofences contained the lowest percentage of smoking events reported between 6 AM and 8:59 AM.

**Figure 2:**
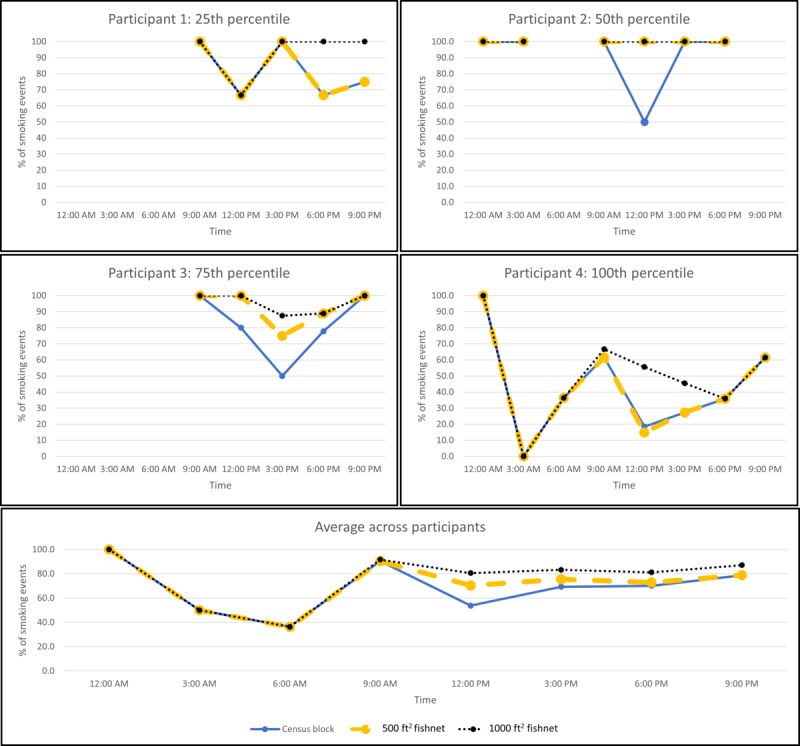
Percent of smoking events captured within each geofence method for 4 participants.

**Figure 3:**
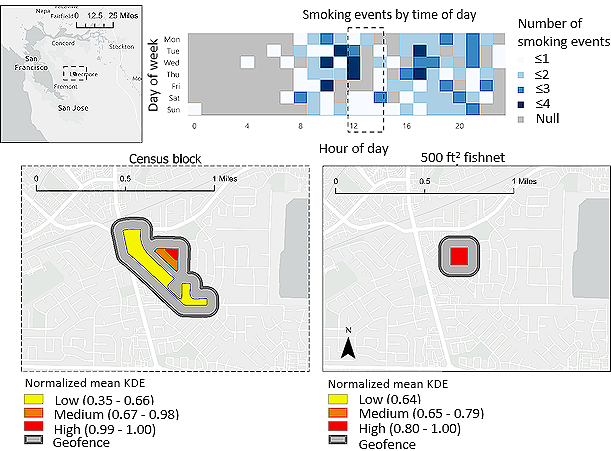
Geofences constructed by the census block versus 500 ft^2^ fishnet grid at noon to 2:59 PM for 1 case. KDE: kernel density estimation.

Across the 4 cases, the 1000 ft^2^ fishnet grid captured the highest percentage of smoking events for each 3-hour interval (Figure 2), both within cases and averaged across cases. Although there was no difference in the percentage of smoking events captured across geofence construction methods between midnight and noon, the constructed geofences looked very different from each other. Figure 3 compares the geofences constructed by the census block and 500 ft^2^ fishnet grid methods for 1 case between noon and 2:59 PM. The census block method’s geofence covers a larger area than the 500 ft^2^ fishnet grid method’s geofence from noon to 2:59 PM, even though they capture the same percentage of smoking events. Further, the census block method generated a wider range of high-risk blocks (normalized mean KDEs between 0.35 and 1.00) across the whole day, whereas the 500 ft^2^ fishnet grid method generated a smaller range of high-risk cells (normalized mean KDEs between 0.64 and 1.00). By depicting the tertiles, we can identify how the distribution of normalized mean KDEs changed spatially across time and method within individuals. For example, Figure 3 tells us that in this period of noon to 2:59 PM, the 500 ft^2^ fishnet grid method created a geofence only around a high-risk cell, whereas the census block method’s geofence encompassed potentially low-risk blocks, yet both capture the same percentage of smoking events for this participant at this time of day. From this, we may visually compare geofence methods and ensure that the highest-risk areas within a geofence are captured, even if the geofence perimeter may differ by method.

**Figure 4:**
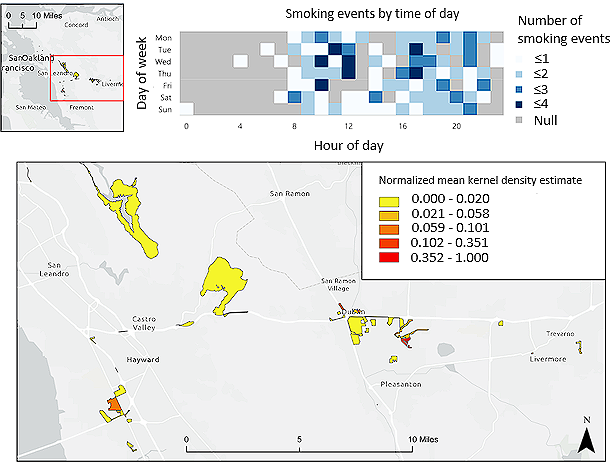
Census blocks with any smoking reports for 100th percentile case across all hours of the day.

Although the 500 ft^2^ fishnet grid method captured a slightly higher percentage of smoking events from 9 AM to 6 PM for the case with the most data, the 100th percentile case (Figure 2), it captured the same percentage of smoking events at all other hours relative to the census block method. To understand why this may have happened, we examined the 100th percentile’s smoking profile. Figure 4 shows where the 100th percentile participant smoked over 26 noncontiguous blocks generated without any thresholds. The blocks are predominantly yellow, representative of normalized mean KDEs below 0.2. This means that many of these blocks contain fewer smoking events. In fact, 73 of 177 (41.8%) smoking events occurred across 2 block areas, while the remaining 104 of 177 (58.2%) smoking events were dispersed across 24 noncontiguous block areas. This is highlighted in census block normalized mean KDE quintiles, in which the bottom 4 quintiles have normalized mean KDEs below 0.35.

As a result, a majority of the 100th percentile case’s zones with smoking events will not result in a geofence to inform intervention delivery because only zones with normalized mean KDEs above 0.3 were retained. For this participant, our method identified that most zones were low-risk relative to other zones, and these low-risk zones would result in a low percentage of smoking events captured in geofences.

## Discussion

### Overview

This study’s objective was to design a spatial approach to identify and construct geofences around person- and time-specific high-risk smoking areas. We collected self-reported, smartphone-delivered surveys on smoking behaviors with passive GPS tracking from young adults who smoke.

Our study found that a kernel density approach for geofence construction could systematically label locations at high risk for smoking. Second, of the 3 methods we used to construct the geofence, the 1000 ft^2^ fishnet grid captured the highest percentage of smoking events within and across the 4 cases. Last, we found that although methods may capture the same percentage of smoking events in the early and late hours of the day, the physical geofences appeared different from each other, which may affect intervention delivery.

### Identified Locations at High Risk for Smoking Through Kernel Density Methods

To our knowledge, this is the first study to examine an individual’s smoking risk profile using KDE methods. Kernel density approaches have already been applied in the tobacco literature to define risk in terms of the tobacco environment (eg, tobacco outlet density) [11,40], and this study demonstrates that KDE methods may also hold value for informing smartphone-based smoking cessation intervention delivery. For our case studies, we found that a normalized mean KDE threshold of 0.3 adequately defined smoking risk.

A KDE approach allowed us to define high-risk locations specific to an individual’s smoking profile. Previous studies have examined risks unspecific to the individual, such as the occurrence of more than 4 smoking events within a geographical region [9]. Four smoking events, however, may be considered high-risk for some individuals and low-risk for others, relative to an individual’s smoking patterns. If we had generated geofences around blocks with smoking events without classifying person-specific, high- and low-risk locations, the intervention delivery would be triggered at locations where the individual rarely smoked relative to other locations. For an ecological momentary intervention, the cumulation of intervention delivery at both efficient and inefficient times and appropriate and inappropriate locations could lead to intervention burden, which may undermine intervention effectiveness and adherence [41]. Prioritizing these high-risk locations and times may be able to reduce intervention burden and improve intervention delivery effectiveness.

The fact that the KDE methods captured most smoking events also highlights their use for people who smoke, especially if they tend to smoke in the same locations. We saw that our methods captured over 50% of smoking events for 3 of the 4 cases. The fourth case, however, was the heaviest smoker based on the self-reported data and frequently smoked in new locations. This may be due to the fact that more frequent smoking indicates greater nicotine dependence and, hence, a more regular need to maintain blood nicotine levels to avoid withdrawal [42]. In addition, this individual may have a more variable activity space than the other 3 cases, resulting in a more spatially dispersed smoking profile. Other strategies than the 1 employed here may be needed to improve intervention delivery for individuals with spatially dispersed smoking profiles.

### Fishnet Grid Geofences Captured More Smoking Events Than Census Block Geofences

We recognized that the UGCoP may result in missing the “true causally relevant” geographic context [25] for smoking cessation geofences, so we generated geofences with 3 different geographical areas—census blocks, 500 ft^2^ fishnet grid cells, and 1000 ft^2^ fishnet grid cells. Of these 3 geographical areas, the 1000 ft^2^ fishnet grid captured the highest percentage of smoking events, followed by the 500 ft^2^ fishnet grid and census blocks. Even the most limited method (census blocks) still effectively captured over half of smoking events. Furthermore, the buffer size of 100 m around adjacent high-risk blocks helped capture some smoking events that would not have been captured otherwise.

Census blocks, however, are often delineated by roads [37] and may misrepresent smoking on the road (eg, the road itself is a very small block below the cell output size, or the smoking event may be forced to one of the adjacent blocks). In the transportation literature, a fishnet grid of half a mile predicted how people traveled better than aggregating to census blocks [43]. Thus, it is possible that the 1000 ft^2^ fishnet grid may have captured more smoking if our cases were smoking in the car, which may have been missed in the 500 ft^2^ fishnet grid and census block methods. Future studies can ask for the smoking location context to discern if this may be the case.

### Selected Method for Effective Intervention Delivery

We found that interventions using the census blocks to create geofences will cover a wider area and may trigger more intervention delivery than interventions using 500 ft^2^ fishnet grid cells for geofence construction. In another study that constructed geofences around locations with more than 4 smoking events, participants had mixed reviews for the frequency of intervention delivery, such that some reported too many alerts and others reported too few [9]. Aligned with the census block method, some participants may want more proactive alerts slightly further away from their usual smoking location, triggered by larger geofences. Future studies may want to compare these different geofence construction methods and their impact on intervention delivery and participant satisfaction.

As researchers seek to define how spaces are categorized as risky or not risky, the modifiable area unit problem needs to be considered as well [44]. As different geofence construction methods may yield different results in intervention delivery, there is a need for a standardized approach for constructing geofences to improve cross-study comparisons. Census blocks are stable for 10 years [37], while fishnet grid cells can shift based on entered parameters [38]. Clear descriptions of all parameters chosen to construct geofences are needed for reproducibility and to help the field develop standards. Depending on the study goal, studies that want to include demographic census data may use census blocks, while those solely interested in optimal intervention delivery may choose fishnet grids. There are also other methods that can weight noncensus data with demographic information [45] that can be further explored.

While a large number of smoking cessation apps are available on the Apple and Google Play app stores, many lack scientific evidence [46,47]. To support individuals in quitting smoking, we need evidence-based interventions that can promote behavior change through positive engagement and personal relevance (eg, appropriate time and place of intervention delivery) [48,49]. Our approach of identifying hot spots of self-reported smoking events and producing geofences that represent high-risk areas for smoking may be helpful to inform future smartphone-based smoking cessation interventions. The proposed approach for generating geofences will be used in an ongoing smoking cessation intervention study with young adults. Given its ability to capture a good percentage of smoking events across compliance levels and the specificity of the constructed geofences, we plan to automate a version of the 1000 ft^2^ fishnet grids to create geofences for participants of an app-based smoking cessation intervention.

### Limitations

Our study has several limitations. First, this was an observational study with individuals who were not ready to quit smoking, which may have impacted compliance to report all smoking events. We attempted to address this issue of compliance by selecting cases to study at quartiles of available data to evaluate the method’s performance for various compliance scenarios. However, EMA compliance may vary significantly between locations, which could potentially affect the results. Second, we assumed smoking reports within 5 minutes of another report were due to technical issues or double reporting based on expert opinion and dropped these reports. Future studies would benefit from including an app feature that sends a follow-up survey to participants after reporting a high volume of smoking events to confirm the number of cigarettes smoked. Third, some census blocks did not rasterize, meaning they were missing a normalized mean KDE due to the block size being smaller than the cell output size. The fishnet grid method captured any of these points that were not rasterized in the census blocks method. Fourth, the risk threshold was determined solely based on the census blocks to allow for a comparison across methods. The census blocks had the greatest variability in normalized mean KDE, and the fishnet grids captured as many or more smoking events than the census blocks. Fifth, our KDE approach used a fixed kernel with a constant bandwidth (which tends to over smooth), whereas adaptive kernels can better capture the scale at which the point pattern process operates [50]. However, we ran KDE for each individual; therefore, the bandwidths were tailored to the individual. Finally, the temporal snapshots of the geofences may not accurately depict the “true” spatiotemporal point-pattern process since we employed a spatial KDE approach. Therefore, future research will employ spatiotemporal point-pattern methods, such as space-time KDE [50] to fully capture the space-time dynamics of the participants and the subsequent creation of geofences for smoking cessation interventions.

### Conclusions

For ecological momentary interventions, it is important to optimize intervention message delivery to minimize intervention burden for participants. Prioritizing intervention delivery to high-risk locations and times may make these interventions relevant for individual participants and consequently improve intervention efficacy. A spatial approach of generating geofences based on high-risk zones (eg, fishnet girds for capturing a greater percentage of events or census blocks for linking spatial data with demographic information) identified through normalized mean KDE surpassing a chosen threshold may assist with prioritizing high-risk locations for intervention delivery, tailored to the needs of an individual participant. By stratifying event occurrence by periods of time, intervention messages can also be appropriately delivered throughout the day. Our study demonstrates that this method can capture a good percentage of smoking events within an urban and suburban environment and illustrates the potential for assessing if it improves person-specific smoking cessation intervention delivery and efficacy. Most importantly, this study highlights that researchers must carefully consider the implications of their chosen geographical unit when designing place-based interventions.

## Appendix

Multimedia Appendix 1 Normalized mean KDE statistics per case.

## Abbreviations

EMA: ecological momentary assessment
KDE: kernel density estimation
mHealth: mobile health
UGCoP: uncertain geographic context problem
UTC: Coordinated Universal Time
