# Supplementary material for: Using Smartphone Survey and GPS Data to Inform Smoking Cessation Intervention Delivery: Case Study
Source: JMIR Mhealth Uhealth. 2023 Jun 16;11:e43990. doi: 10.2196/43990 (PMC10337446; doi:10.2196/43990)
Supplement: Multimedia Appendix 1 [file mhealth_v11i1e43990_app1.docx]

| **Table S1.**  Normalized mean KDE statistics per case. | | | | | | | | | | |
| --- | --- | --- | --- | --- | --- | --- | --- | --- | --- | --- |
|  |  | Top 100 | | | Top 70 | | | | | |
| Participant Percentile | Zone Type | N Zones | (Min, Max) | Mean (SD) | N Zones | (Min, Max) | Mean (SD) | Low Risk | Medium Risk | High Risk |
| 25^th^ | Census blocks | 8.00 | (0.02, 1.00) | 0.55 (0.35) | 5.00 | (0.62, 1.00) | 0.8 (0.14) | 0.62-0.68 | 0.68-0.91 | 0.91-1.00 |
| 25^th^ | 500 ft^2^ fishnet | 6.00 | (0.13, 1.00) | 0.53 (0.35) | 3.00 | (0.66, 1.00) | 0.86 (0.14) | 0.66 | 0.66-0.92 | 0.92-1.00 |
| 25^th^ | 1000 ft^2^ fishnet | 5.00 | (0.16, 1.00) | 0.64 (0.33) | 4.00 | (0.34, 1.00) | 0.76 (0.25) | 0.34-0.84 | 0.84-0.85 | 0.85-1.00 |
| 50^th^ | Census blocks | 10.00 | (0.25, 1.00) | 0.58 (0.24) | 9.00 | (0.32, 1.00) | 0.61 (0.23) | 0.32-0.46 | 0.46-0.57 | 0.57-1.00 |
| 50^th^ | 500 ft^2^ fishnet | 10.00 | (0.47, 1.00) | 0.67 (0.22) | 10.00 | (0.47, 1.00) | 0.67 (0.22) | 0.47-0.49 | 0.49-0.86 | 0.86-1.00 |
| 50^th^ | 1000 ft^2^ fishnet | 9.00 | (0.35, 1.00) | 0.6 (0.25) | 9.00 | (0.35, 1.00) | 0.6 (0.25) | 0.35-0.42 | 0.42-0.48 | 0.48-1.00 |
| 75^th^ | Census blocks | 12.00 | (0.04, 1.00) | 0.43 (0.33) | 6.00 | (0.32, 1.00) | 0.71 (0.23) | 0.33-0.53 | 0.53-0.78 | 0.78-1.00 |
| 75^th^ | 500 ft^2^ fishnet | 11.00 | (0.05, 1.00) | 0.38 (0.32) | 4.00 | (0.32, 1.00) | 0.74 (0.27) | 0.32-0.70 | 0.70-0.95 | 0.95-1.00 |
| 75^th^ | 1000 ft^2^ fishnet | 7.00 | (0.05, 1.00) | 0.41 (0.36) | 3.00 | (0.41, 1.00) | 0.77 (0.26) | 0.41 | 0.41-0.90 | 0.90-1.00 |
| 100^th^ | Census blocks | 58.00 | (0.00, 1.00) | 0.08 (0.2) | 4.00 | (0.35, 1.00) | 0.75 (0.27) | 0.35-0.66 | 0.67-0.98 | 0.99-1.00 |
| 100^th^ | 500 ft^2^ fishnet | 64.00 | (0.01, 1.00) | 0.09 (0.2) | 4.00 | (0.64, 1.00) | 0.84 (0.14) | 0.64 | 0.65-0.79 | 0.80-1.00 |
| 100^th^ | 1000 ft^2^ fishnet | 54.00 | (0.01, 1.00) | 0.09 (0.19) | 5.00 | (0.33, 1.00) | 0.68 (0.23) | 0.33-0.59 | 0.60-0.84 | 0.85-1.00 |

Top 100 denotes all KDE values are retained. Top 70 indicates that normalized mean KDEs greater than 0.3 are retained.
